# Supplementary material for: An Analysis of JADE2 in Non-Small Cell Lung Cancer (NSCLC)
Source: Biomedicines. 2023 Sep 19;11(9):2576. doi: 10.3390/biomedicines11092576 (PMC10526426; doi:10.3390/biomedicines11092576)
Supplement: Supplementary file 1 [file biomedicines-11-02576-s001.zip › Supplementary Figure Legends.pdf]

Supplementary Figure Legends

Supplementary Figure S1. Expression of JADE family member's mRNA in LUAD. Using LCE and cProSite, the expression of the JADE family at the mRNA and protein level in LUAD was assessed for (A) JADE1 mRNA; (B) JADE1 protein; (C) JADE2 mRNA; (D) JADE2 protein; (E) JADE3 mRNA and (F) JADE3 protein.

Supplementary Figure S2. Expression of JADE family member's mRNA in LUSC. Using LCE and cProSite, the expression of the JADE family at the mRNA and protein level in LUSC was assessed for (A) JADE1 mRNA; (B) JADE1 protein; (C) JADE2 mRNA; (D) JADE2 protein; (E) JADE3 mRNA and (F) JADE3 protein.

Supplementary Figure S3. **Pan-Cancer analysis of JADE1 mRNA**. Using GEPIA2 a pan-cancer analysis of JADE1 mRNA was conducted that also incorporated GTEx normal tissue expression.

Supplementary Figure S4. **Pan-Cancer analysis of JADE2 mRNA**. Using GEPIA2 a pan-cancer analysis of JADE2 mRNA was conducted that also incorporated GTEx normal tissue expression.

Supplementary Figure S5. **Pan-Cancer analysis of JADE3 mRNA**. Using GEPIA2 a pan-cancer analysis of JADE3 mRNA was conducted that also incorporated GTEx normal tissue expression.

Supplementary Figure S6. **Pan-Cancer analysis of JADE family mRNA gene signature**. Using GEPIA2 a pan-cancer analysis of the combined JADE family gene mRNA signature mRNA was conducted that also incorporated GTEx normal tissue expression.

Supplementary Figure S7. Identification of Ornidazole as a candidate drug for repurposing in NSCLC. DepMap was used to analyze cell lines restricted solely to lung cancer, and identified ornidazole as a candidate drug linked to the expression of the mRNA for JADE2.

Supplementary Figure S8. Prognostic value of JADE2 mRNA as measured by PFS in NSCLC. The prognostic value of *JADE2* mRNA expression was assessed for progression free survival (PFS) using KM-Plotter . Higher expression of the mRNA for *JADE2* was associated with better PFS overall (A); which when stratified by tumor histology was limited to the LUAD subtype only (B); whilst no difference in PFS was observed for LUSC (C). $p<0.05$ was considered to be significant.
