# Supplementary material for: An Analysis of JADE2 in Non-Small Cell Lung Cancer (NSCLC)
Source: Biomedicines. 2023 Sep 19;11(9):2576. doi: 10.3390/biomedicines11092576 (PMC10526426; doi:10.3390/biomedicines11092576)
Supplement: Supplementary file 1 [file biomedicines-11-02576-s001.zip › Supplementary Figure S8.pdf]

(A) All Histologies

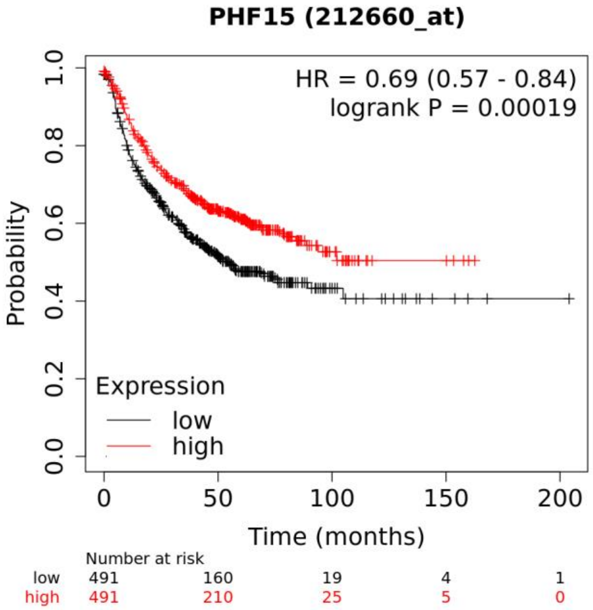

Low expression cohort (months)
13.54

High expression cohort (months)
22.53

(B) LUAD

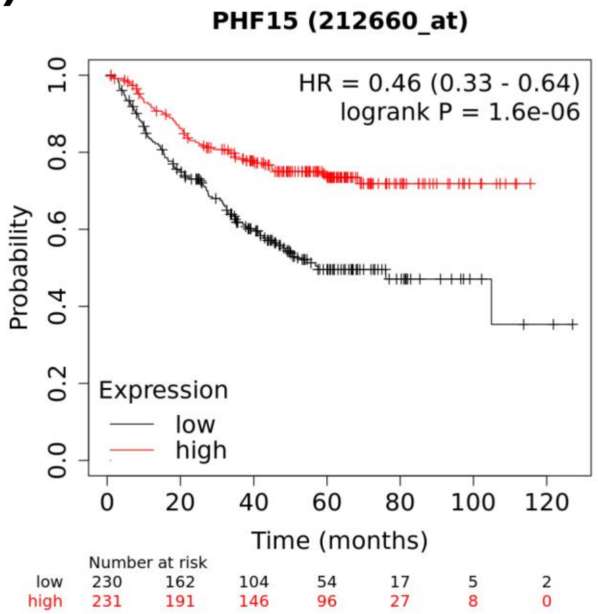

Low expression cohort (months)
20.07

High expression cohort (months)
45.3

(C) LUSC

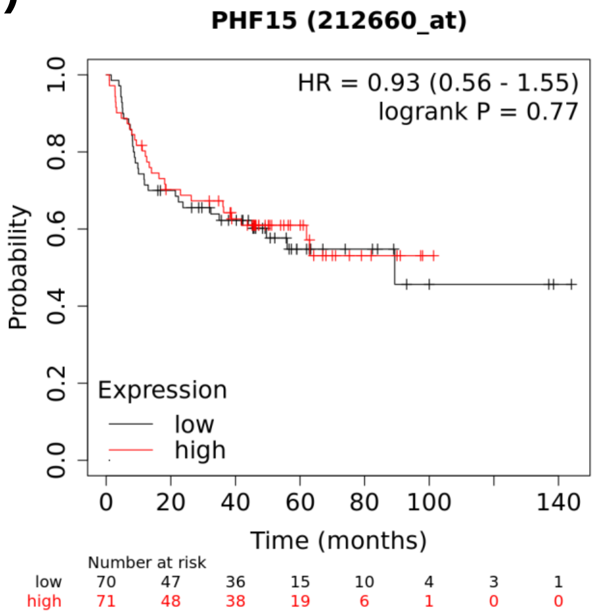

Low expression cohort (months)
10

High expression cohort (months)
14
