# Supplementary material for: An Analysis of JADE2 in Non-Small Cell Lung Cancer (NSCLC)
Source: Biomedicines. 2023 Sep 19;11(9):2576. doi: 10.3390/biomedicines11092576 (PMC10526426; doi:10.3390/biomedicines11092576)
Supplement: Supplementary file 1 [file biomedicines-11-02576-s001.zip › Supplementary Table S1 MuTarget LUAD.pdf]

Supplementary Table S1. Mutated genes associated with significantly altered JADE2 expression in LUAD as determined using MuTarget.

Mutation of	Mean expression (mutant)	Mean expression (wild)	Number of mutant	Number of wild	FC (mutant/wild)	Direction	p-value
SLC22A25	853.08	1674.28	12	496	1.96	down	3.60E-05
KCNT1	934.19	1678.32	16	492	1.79	down	4.06E-05
HECW2	1047.79	1678.47	19	489	1.61	down	1.21E-04
KRT34	893.5	1673.31	12	496	1.89	down	2.11E-04
CNTN4	1032.11	1677.76	18	490	1.61	down	2.33E-04
SYCP2	1092.18	1674.37	17	491	1.54	down	5.07E-04
TOX2	1010.87	1674.48	15	493	1.67	down	5.41E-04
ADGRB1	1095.59	1674.25	17	491	1.54	down	7.67E-04
PIK3AP1	1123.22	1674.42	18	490	1.49	down	1.01E-03
CAND1	1004.27	1669.29	11	497	1.67	down	1.37E-03
CADPS	1156.1	1676.39	21	487	1.45	down	1.46E-03
RNF133	979.91	1669.82	11	497	1.69	down	1.56E-03
DSG2	1015.36	1669.04	11	497	1.64	down	1.83E-03
NCAM2	1165.13	1678.11	23	485	1.45	down	1.86E-03
MAST1	995.27	1669.48	11	497	1.67	down	1.95E-03
ATP12A	1107.53	1671.54	15	493	1.52	down	2.22E-03
UBTFL1	1095.83	1668.41	12	496	1.52	down	2.49E-03
EYA4	1158.35	1675.24	20	488	1.45	down	2.56E-03
TCF20	1147.27	1670.33	15	493	1.45	down	3.13E-03
PABPC5	1106.36	1670.43	14	494	1.52	down	3.23E-03
ATOH1	1149.67	1670.26	15	493	1.45	down	3.31E-03
OR4D5	1074	1668.94	12	496	1.56	down	3.43E-03
TCERG1L	1093.46	1669.63	13	495	1.54	down	3.44E-03
PRAMEF11	1078.31	1670.03	13	495	1.54	down	3.54E-03
RREB1	1115.21	1670.18	14	494	1.49	down	3.58E-03

ZNF578	1156.25	1671.1	16	492	1.45	down	3.61E-03
ADCYAP1R1	1078.75	1668.82	12	496	1.54	down	3.65E-03
TMTC2	1070.08	1669.03	12	496	1.56	down	3.83E-03
ATP8B3	1077.75	1668.85	12	496	1.54	down	3.98E-03
REG3G	1130.53	1673.04	17	491	1.47	down	3.99E-03
ZNF267	1149.93	1669.2	14	494	1.45	down	4.06E-03
TCF4	1147.8	1670.31	15	493	1.45	down	4.09E-03
NUTM1	1139.87	1670.56	15	493	1.47	down	4.13E-03
KRT6B	1116.4	1671.27	15	493	1.49	down	4.24E-03
SLC6A18	1137.59	1672.8	17	491	1.47	down	4.29E-03
MAATS1	1161.28	1673.02	18	490	1.45	down	4.31E-03
CACHD1	1049.18	1668.29	11	497	1.59	down	4.54E-03
ATP10A	1132.44	1671.88	16	492	1.47	down	4.74E-03
ITGAV	1109	1669.22	13	495	1.52	down	4.81E-03
ZZEF1	1142.56	1673.71	18	490	1.47	down	5.38E-03
IFI44L	1134.43	1669.64	14	494	1.47	down	5.38E-03
CCDC144A	1036.91	1668.56	11	497	1.61	down	5.46E-03
DCLK1	1136.88	1671.73	16	492	1.47	down	5.46E-03
SLC17A4	1118.67	1671.2	15	493	1.49	down	5.82E-03
MAGEB1	1128.07	1669.82	14	494	1.47	down	6.39E-03
USH1C	1115.23	1669.06	13	495	1.49	down	6.98E-03
KLB	1160.07	1669.94	15	493	1.45	down	7.09E-03
IRX1	1141.87	1670.49	15	493	1.47	down	7.28E-03
MCM10	1067.09	1667.9	11	497	1.56	down	7.46E-03
SCYL2	1115.55	1666.82	11	497	1.49	down	7.74E-03
SFSWAP	1120.33	1667.82	12	496	1.49	down	8.17E-03
PHF20L1	1158.13	1670	15	493	1.45	down	8.36E-03
EIF4G3	1126.77	1668.76	13	495	1.47	down	8.88E-03
REM1	1133.92	1668.57	13	495	1.47	down	8.91E-03

GPC4	1107.85	1669.25	13	495	1.52	down	8.93E-03
HEG1	1068	1667.88	11	497	1.56	down	9.27E-03
PIK3C2G	1154.65	1672.21	17	491	1.45	down	9.77E-03
SPATA31A7	1068.55	1667.86	11	497	1.56	down	9.81E-03
SMC2	1147.23	1668.22	13	495	1.45	down	9.93E-03
