# Supplementary material for: An Analysis of JADE2 in Non-Small Cell Lung Cancer (NSCLC)
Source: Biomedicines. 2023 Sep 19;11(9):2576. doi: 10.3390/biomedicines11092576 (PMC10526426; doi:10.3390/biomedicines11092576)
Supplement: Supplementary file 1 [file biomedicines-11-02576-s001.zip › Supplementary Table S2 MuTarget LUSC.pdf]

Supplementary Table S2. Mutated genes associated with significantly altered JADE2 expression in LUSC as determined using MuTarget.

Mutation of	Mean expression (mutant)	Mean expression (wild)	Number of mutant	Number of wild	FC (mutant/wild)	Direction	p-value
TEP1	1184.76	2038.64	17	471	1.72	down	6.55E-04
ZNF800	4156.3	1963.97	10	478	2.12	up	6.74E-04
HECW1	1248.88	2062.23	32	456	1.64	down	8.54E-04
AMER1	1007.05	2049.48	19	469	2.04	down	9.90E-04
MYOM3	956.13	2042.28	15	473	2.13	down	1.67E-03
TYK2	852.64	2035.56	11	477	2.38	down	1.95E-03
POLA1	914.31	2038.85	13	475	2.22	down	2.40E-03
SMARCA4	1098.35	2041.76	17	471	1.85	down	2.57E-03
CWF19L2	993.4	2041.1	15	473	2.04	down	2.90E-03
DOPEY2	3772	1964.45	12	476	1.92	up	3.55E-03
IGSF11	3029.56	1974.3	16	472	1.53	up	3.92E-03
KCTD16	3690.53	1955.57	15	473	1.89	up	4.42E-03
AGMO	1070.75	2040.7	16	472	1.92	down	6.63E-03
PADI1	1146	2028.8	11	477	1.79	down	6.76E-03
MYH11	1176.89	2042.6	19	469	1.72	down	6.76E-03
CHD3	961.15	2037.57	13	475	2.13	down	7.01E-03
DNAH3	1342.56	2073.29	43	445	1.54	down	7.60E-03
OR6K3	3266.78	1960.72	18	470	1.67	up	8.44E-03
TECRL	1176.47	2038.94	17	471	1.72	down	8.73E-03
KIF17	912.4	2031.84	10	478	2.22	down	9.26E-03
PRSS16	3240.5	1983.13	10	478	1.63	up	9.48E-03
EPHA3	1397.91	2056.1	35	453	1.47	down	9.98E-03
