# Supplementary material for: An Analysis of JADE2 in Non-Small Cell Lung Cancer (NSCLC)
Source: Biomedicines. 2023 Sep 19;11(9):2576. doi: 10.3390/biomedicines11092576 (PMC10526426; doi:10.3390/biomedicines11092576)
Supplement: Supplementary file 1 [file biomedicines-11-02576-s001.zip › Supplementary Table S3 TIIL LUAD.pdf]

Supplementary Table S3: TIMER2 analysis of Immune Cell infiltrations associated with JADE2 mRNA expression in LUAD

Infiltrates	rho	p	adj.p
**B Cells**			
B cell memory_CIBERSORT	0.166845	**0.000198**	0.001307
B cell memory_CIBERSORT-ABS	0.2148	**1.48E-06**	1.71E-05
B cell memory_XCELL	-0.06192	0.169884	0.297172
B cell naive_CIBERSORT	-0.1131	**0.011974**	0.039046
B cell naive_CIBERSORT-ABS	-0.03625	0.421926	0.565079
B cell naive_XCELL	0.099242	**0.027569**	0.072233
B cell plasma_CIBERSORT	-0.21243	**1.94E-06**	2.20E-05
B cell plasma_CIBERSORT-ABS	-0.05433	0.228538	0.369603
B cell plasma_XCELL	-0.21541	**1.38E-06**	1.63E-05
B cell_EPIC	0.094217	**0.036501**	0.09202
B cell_MCPCOUNTER	0.150709	**0.000788**	0.004111
B cell_QUANTISEQ	0.160785	**0.000338**	0.002112
B cell_TIMER	0.106972	**0.017503**	0.051555
B cell_XCELL	0.123861	**0.005891**	0.022513
Class-switched memory B cell_XCELL	0.259172	**5.22E-09**	1.04E-07
**CD8+ T Cells**			
T cell CD8+ central memory_XCELL	0.090543	**0.044495**	0.106653
T cell CD8+ effector memory_XCELL	0.135344	**0.002601**	0.009544
T cell CD8+ naive_XCELL	-0.18011	**5.78E-05**	0.000309
T cell CD8+_CIBERSORT	-0.03111	0.490752	0.633229
T cell CD8+_CIBERSORT-ABS	0.208726	**2.95E-06**	2.13E-05
T cell CD8+_EPIC	0.081854	0.069388	0.153344
T cell CD8+_MCPCOUNTER	0.070287	0.119092	0.236999
T cell CD8+_QUANTISEQ	0.149393	**0.000877**	0.003473
T cell CD8+_TIMER	0.246549	**2.92E-08**	3.65E-07
T cell CD8+_XCELL	0.134134	**0.002843**	0.010065
**CD4+ T Cells**			
T cell CD4+ (non-regulatory)_QUANTISEQ	0.090543	**0.044495**	0.106653
T cell CD4+ (non-regulatory)_XCELL	0.135344	**0.002601**	0.009544
T cell CD4+ central memory_XCELL	-0.18011	**5.78E-05**	0.000309
T cell CD4+ effector memory_XCELL	-0.03111	0.490752	0.633229
T cell CD4+ memory activated_CIBERSORT	0.208726	**2.95E-06**	2.13E-05
T cell CD4+ memory activated_CIBERSORT-ABS	0.081854	0.069388	0.153344
T cell CD4+ memory resting_CIBERSORT	0.070287	0.119092	0.236999
T cell CD4+ memory resting_CIBERSORT-ABS	0.149393	**0.000877**	0.003473
T cell CD4+ memory_XCELL	0.246549	**2.92E-08**	3.65E-07
T cell CD4+ naïve_CIBERSORT	0.134134	**0.002843**	0.010065
T cell CD4+ naïve_CIBERSORT-ABS	0.090543	**0.044495**	0.106653
T cell CD4+ naïve_XCELL	0.135344	**0.002601**	0.009544
T cell CD4+ Th1_XCELL	-0.18011	**5.78E-05**	0.000309

T cell CD4+ Th2_XCELL	-0.03111	0.490752	0.633229
T cell CD4+_EPIC	0.208726	**2.95E-06**	2.13E-05
T cell CD4+_TIMER	0.081854	0.069388	0.153344
**Macrophages**			
Macrophage M0_CIBERSORT	-0.10907	**0.015398**	0.036611
Macrophage M0_CIBERSORT-ABS	-0.02392	0.596202	0.679621
Macrophage M1_CIBERSORT	-0.02472	0.584012	0.667443
Macrophage M1_CIBERSORT-ABS	0.155166	**0.000545**	0.001913
Macrophage M1_QUANTISEQ	0.349864	**1.22E-15**	4.29E-14
Macrophage M1_XCELL	0.039693	0.379167	0.489247
Macrophage M2_CIBERSORT	0.109031	**0.015437**	0.036611
Macrophage M2_CIBERSORT-ABS	0.382891	**1.16E-18**	6.34E-17
Macrophage M2_QUANTISEQ	0.289641	**5.54E-11**	7.38E-10
Macrophage M2_TIDE	-0.20586	**4.05E-06**	2.15E-05
Macrophage M2_XCELL	0.087414	0.052417	0.10414
Macrophage_EPIC	0.13754	**0.002208**	0.006495
Macrophage_TIMER	0.085324	0.058339	0.111893
Macrophage_XCELL	0.096552	**0.032084**	0.068023
Macrophage/Monocyte_MCPCOUNTER	0.175985	**8.56E-05**	0.000347
**Neutrophils**			
Neutrophil_CIBERSORT	0.005188	0.90852	0.948193
Neutrophil_CIBERSORT-ABS	0.052623	0.243505	0.369816
Neutrophil_MCPCOUNTER	0.330682	**4.82E-14**	7.68E-13
Neutrophil_QUANTISEQ	0.227347	**3.36E-07**	2.68E-06
Neutrophil_TIMER	0.248544	**2.24E-08**	1.98E-07
Neutrophil_XCELL	-0.09396	**0.037008**	0.081896
**Dendritic Cells**			
Myeloid dendritic cell activated_CIBERSORT	0.091392	**0.042527**	0.116728
Myeloid dendritic cell activated_CIBERSORT-ABS	0.139556	**0.001896**	0.008116
Myeloid dendritic cell activated_XCELL	0.235795	**1.18E-07**	1.23E-06
Myeloid dendritic cell resting_CIBERSORT	0.127863	**0.004462**	0.017242
Myeloid dendritic cell resting_CIBERSORT-ABS	0.185883	**3.28E-05**	0.000211
Myeloid dendritic cell_MCPCOUNTER	0.298088	**1.42E-11**	2.82E-10
Myeloid dendritic cell_QUANTISEQ	-0.11106	**0.013617**	0.045928
Myeloid dendritic cell_TIMER	0.326905	**9.65E-14**	2.96E-12
Myeloid dendritic cell_XCELL	0.305082	**4.44E-12**	9.30E-11
Plasmacytoid dendritic cell_XCELL	-0.10327	**0.021833**	0.068422
