# Supplementary material for: An Analysis of JADE2 in Non-Small Cell Lung Cancer (NSCLC)
Source: Biomedicines. 2023 Sep 19;11(9):2576. doi: 10.3390/biomedicines11092576 (PMC10526426; doi:10.3390/biomedicines11092576)
Supplement: Supplementary file 1 [file biomedicines-11-02576-s001.zip › Supplementary Table S4 TIIL LUSC.pdf]

Supplementary Table S4. TIMER2 analysis of Immune Cell infiltrations associated with JADE2 expression in LUSC

Infiltrates	rho	p	adj.p
**B Cells**			
B cell memory_CIBERSORT	0.02508	0.584787	0.716066
B cell memory_CIBERSORT-ABS	0.039844	0.38525	0.538811
B cell memory_XCELL	0.021477	0.639861	0.759793
B cell naive_CIBERSORT	0.054875	0.231601	0.371552
B cell naive_CIBERSORT-ABS	0.101691	**0.026358**	0.070289
B cell naive_XCELL	0.176781	**0.000104**	0.000742
B cell plasma_CIBERSORT	-0.20345	**7.51E-06**	7.27E-05
B cell plasma_CIBERSORT-ABS	-0.09262	**0.04319**	0.106205
B cell plasma_XCELL	-0.08961	**0.050463**	0.120149
B cell_EPIC	0.045308	0.32342	0.465353
B cell_MCPCOUNTER	0.032105	0.484219	0.61947
B cell_QUANTISEQ	0.153794	**0.000751**	0.004024
B cell_TIMER	-0.0225	0.624005	0.750467
B cell_XCELL	0.125384	**0.006105**	0.023185
Class-switched memory B cell_XCELL	0.188575	**3.40E-05**	0.000279
**CD8+ T Cells**			
T cell CD8+ central memory_XCELL	0.048659	0.288885	0.431171
T cell CD8+ effector memory_XCELL	0.131983	**0.003882**	0.01327
T cell CD8+ naive_XCELL	-0.10274	**0.024835**	0.065789
T cell CD8+_CIBERSORT	0.060393	0.187926	0.321241
T cell CD8+_CIBERSORT-ABS	0.172594	**0.000152**	0.000714
T cell CD8+_EPIC	-0.08108	0.076897	0.167167
T cell CD8+_MCPCOUNTER	0.046617	0.309622	0.448728
T cell CD8+_QUANTISEQ	0.055691	0.224728	0.363933
T cell CD8+_TIMER	0.084692	0.064579	0.145941
T cell CD8+_XCELL	0.051743	0.259369	0.399028
**CD4+ T Cells**			
T cell CD4+ (non-regulatory)_QUANTISEQ	0.048659	0.288885	0.431171
T cell CD4+ (non-regulatory)_XCELL	0.131983	**0.003882**	0.01327
T cell CD4+ central memory_XCELL	-0.10274	**0.024835**	0.065789
T cell CD4+ effector memory_XCELL	0.060393	0.187926	0.321241
T cell CD4+ memory activated_CIBERSORT	0.172594	**0.000152**	0.000714
T cell CD4+ memory activated_CIBERSORT-ABS	-0.08108	0.076897	0.167167
T cell CD4+ memory resting_CIBERSORT	0.046617	0.309622	0.448728
T cell CD4+ memory resting_CIBERSORT-ABS	0.055691	0.224728	0.363933
T cell CD4+ memory_XCELL	0.084692	0.064579	0.145941
T cell CD4+ naïve_CIBERSORT	0.051743	0.259369	0.399028
T cell CD4+ naïve_CIBERSORT-ABS	0.048659	0.288885	0.431171
T cell CD4+ naïve_XCELL	0.131983	**0.003882**	0.01327
T cell CD4+ Th1_XCELL	-0.10274	0.024835	0.065789

T cell CD4+ Th2_XCELL	0.060393	0.187926	0.321241
T cell CD4+_EPIC	0.172594	**0.000152**	0.000714
T cell CD4+_TIMER	-0.08108	0.076897	0.167167
**Macrophages**			
Macrophage M0_CIBERSORT	0.138445	**0.002443**	0.007012
Macrophage M0_CIBERSORT-ABS	0.231153	**3.32E-07**	2.19E-06
Macrophage M1_CIBERSORT	0.112328	**0.014104**	0.033714
Macrophage M1_CIBERSORT-ABS	0.200352	**1.04E-05**	5.28E-05
Macrophage M1_QUANTISEQ	0.084314	0.065783	0.125302
Macrophage M1_XCELL	0.125232	**0.006168**	0.015748
Macrophage M2_CIBERSORT	-0.05526	0.228339	0.334971
Macrophage M2_CIBERSORT-ABS	0.123764	**0.006802**	0.017149
Macrophage M2_QUANTISEQ	0.184143	**5.21E-05**	0.000225
Macrophage M2_TIDE	-0.1682	**0.000224**	0.000846
Macrophage M2_XCELL	-0.04097	0.37199	0.483104
Macrophage_EPIC	0.125234	**0.006167**	0.015748
Macrophage_TIMER	0.025488	0.578691	0.665162
Macrophage_XCELL	0.075614	0.099052	0.176878
Macrophage/Monocyte_MCPCOUNTER	0.196562	**1.53E-05**	7.36E-05
**Neutrophils**			
Neutrophil_CIBERSORT	-0.17709	**0.000101**	0.000496
Neutrophil_CIBERSORT-ABS	-0.14692	**0.001292**	0.00454
Neutrophil_MCPCOUNTER	0.195444	**1.72E-05**	9.77E-05
Neutrophil_QUANTISEQ	-0.03395	0.459447	0.616898
Neutrophil_TIMER	0.040949	0.372197	0.51718
Neutrophil_XCELL	-0.15688	**0.000585**	0.002218
**Dendritic Cells**			
Myeloid dendritic cell activated_CIBERSORT	-0.13947	**0.002265**	0.009489
Myeloid dendritic cell activated_CIBERSORT-ABS	-0.10888	**0.017371**	0.05667
Myeloid dendritic cell activated_XCELL	0.168034	**0.000227**	0.001257
Myeloid dendritic cell resting_CIBERSORT	-0.03695	0.420743	0.581444
Myeloid dendritic cell resting_CIBERSORT-ABS	-3.54E-06	0.999938	0.999938
Myeloid dendritic cell_MCPCOUNTER	0.141675	**0.001924**	0.008145
Myeloid dendritic cell_QUANTISEQ	0.00617	0.893076	0.937847
Myeloid dendritic cell_TIMER	0.129216	**0.004705**	0.017962
Myeloid dendritic cell_XCELL	0.026049	0.570359	0.711607
Plasmacytoid dendritic cell_XCELL	0.154546	**0.000707**	0.003562
