# Supplementary figures and images for: An Analysis of JADE2 in Non-Small Cell Lung Cancer (NSCLC)
Source: Biomedicines. 2023 Sep 19;11(9):2576. doi: 10.3390/biomedicines11092576 (PMC10526426; doi:10.3390/biomedicines11092576)

Tumor

Normal

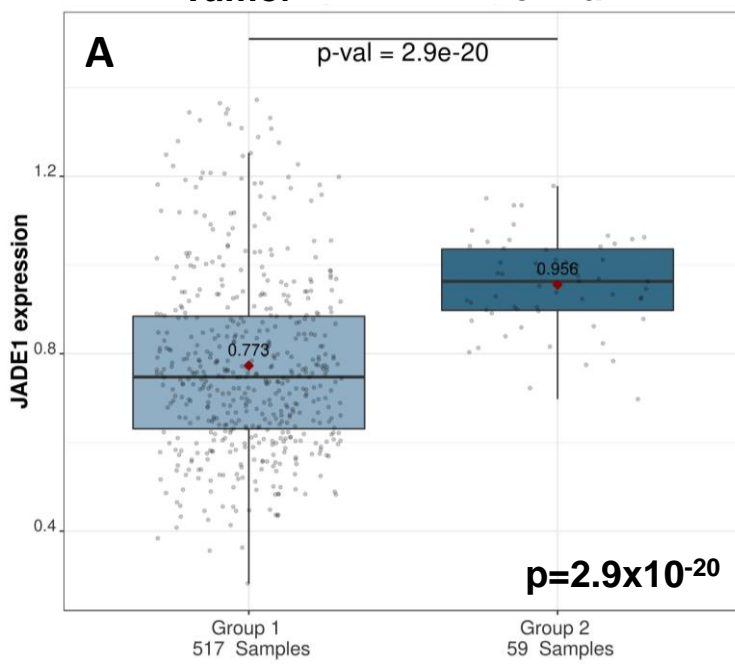

Tumor

Normal

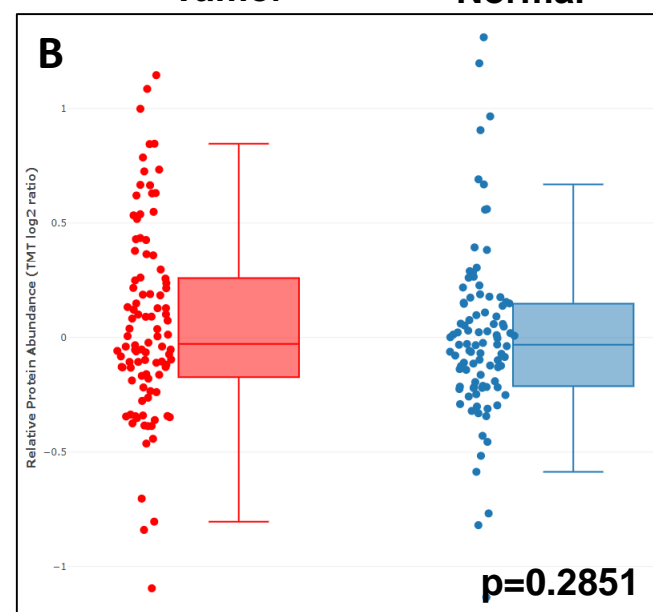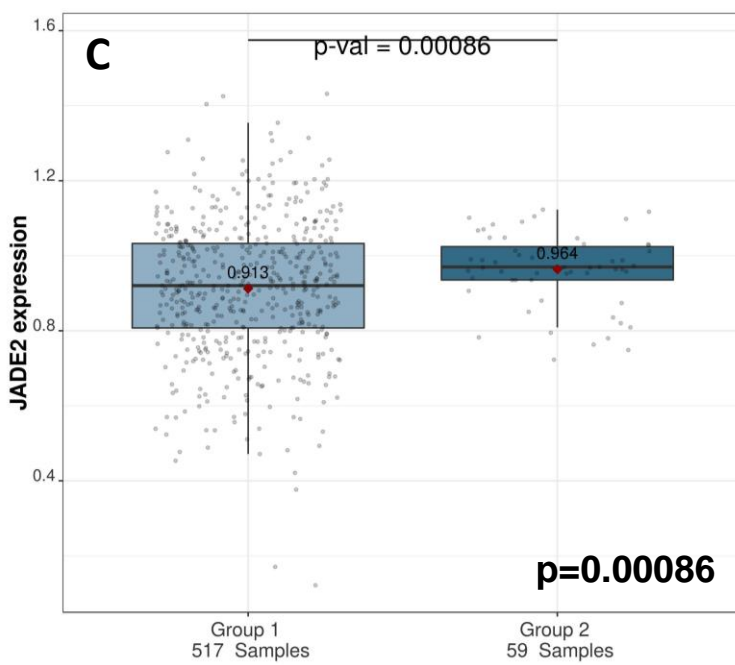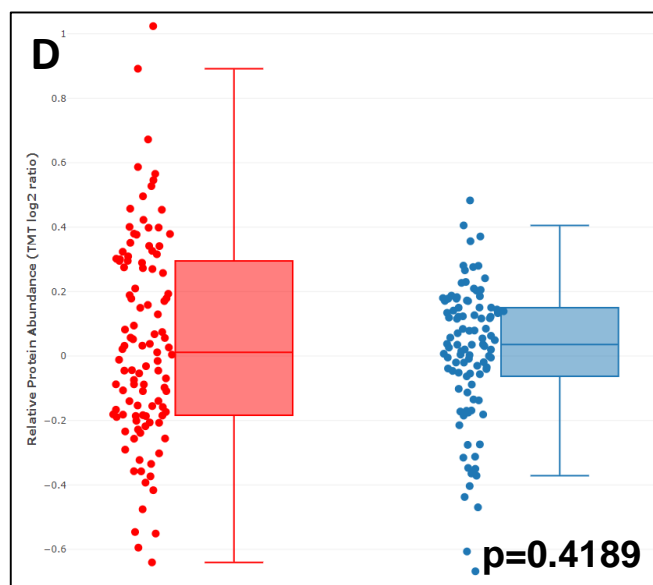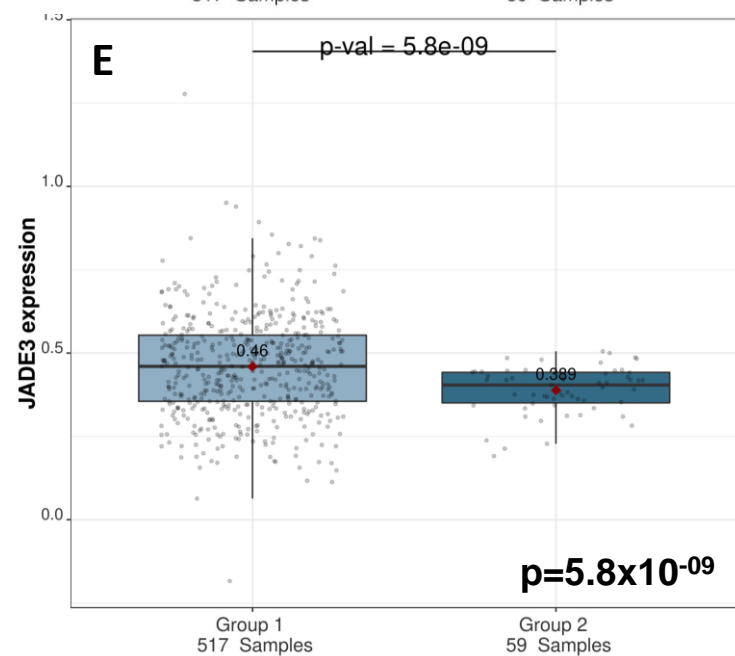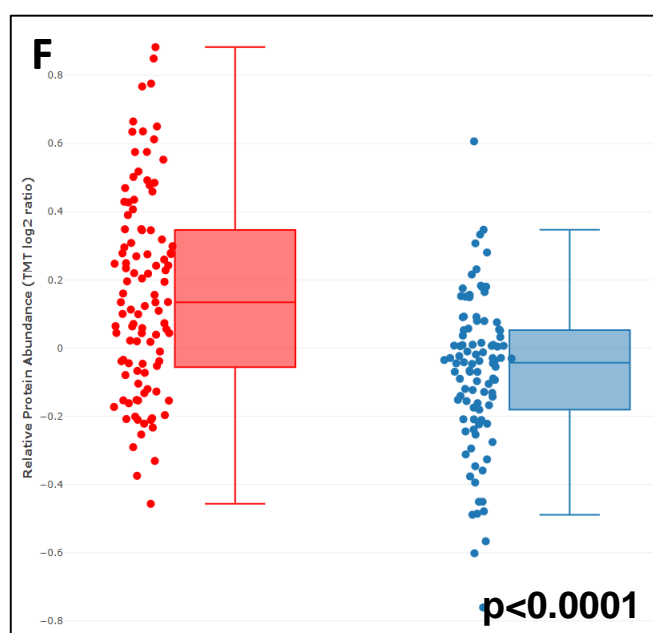

Supplement: Supplementary file 1 [file biomedicines-11-02576-s001.zip › Supplementary Figure S1.pdf]

**Tumor**

**Normal**

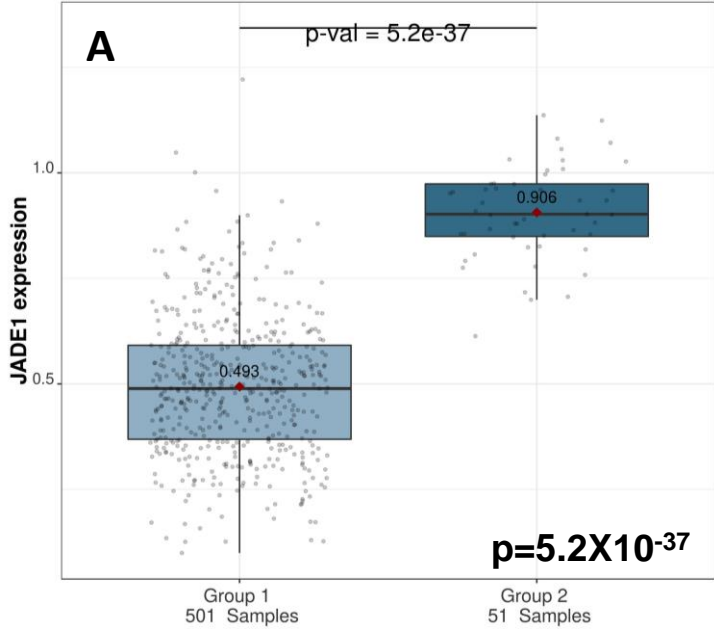

**Tumor**

**Normal**

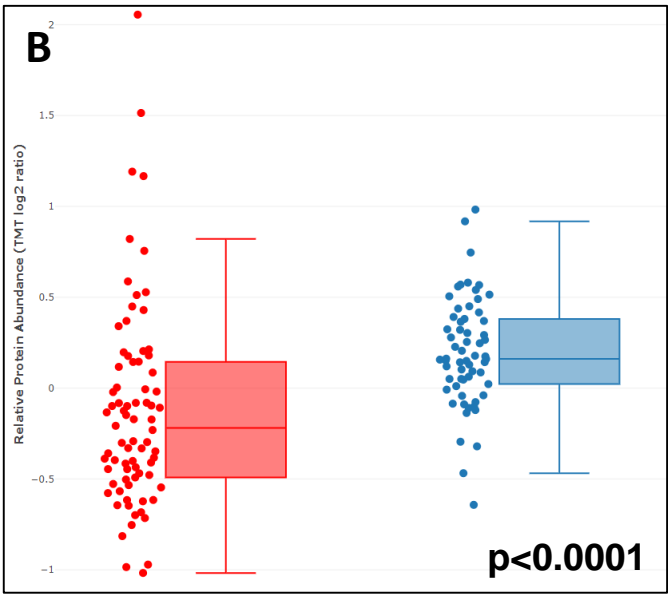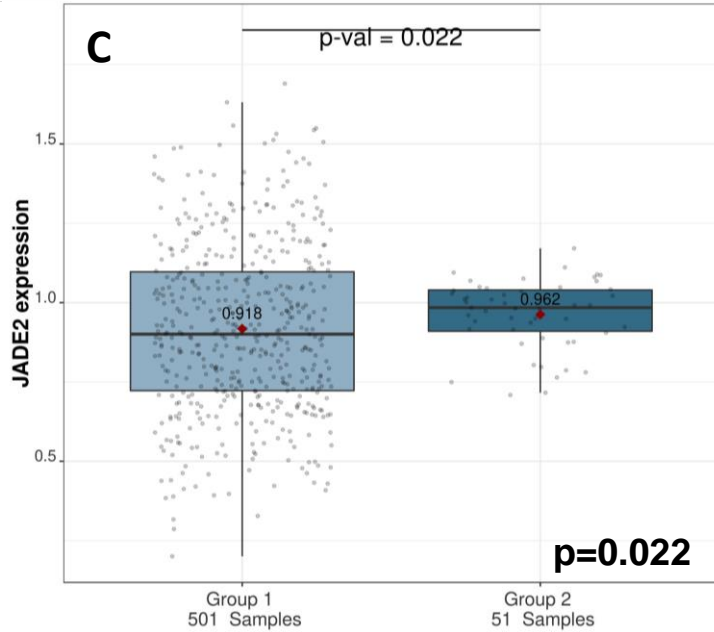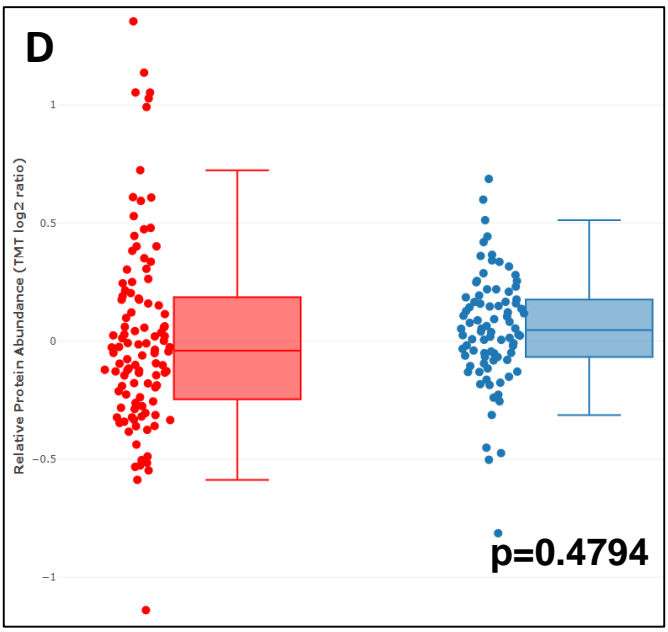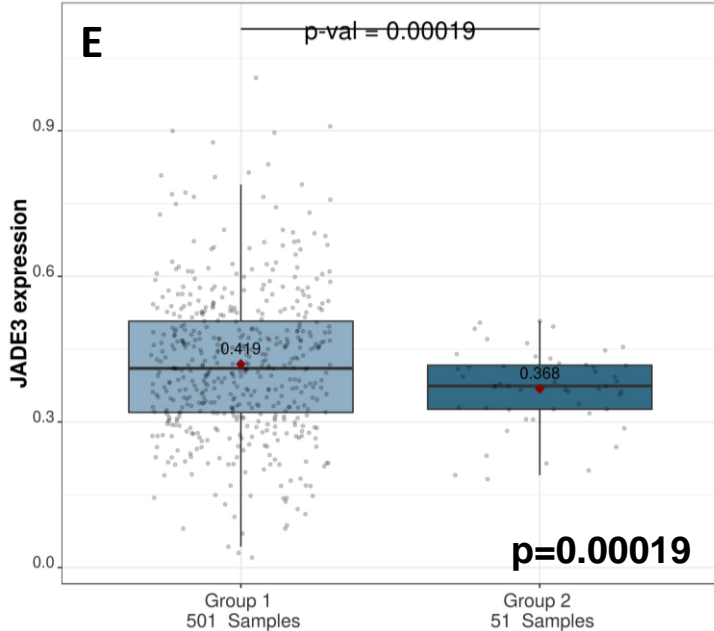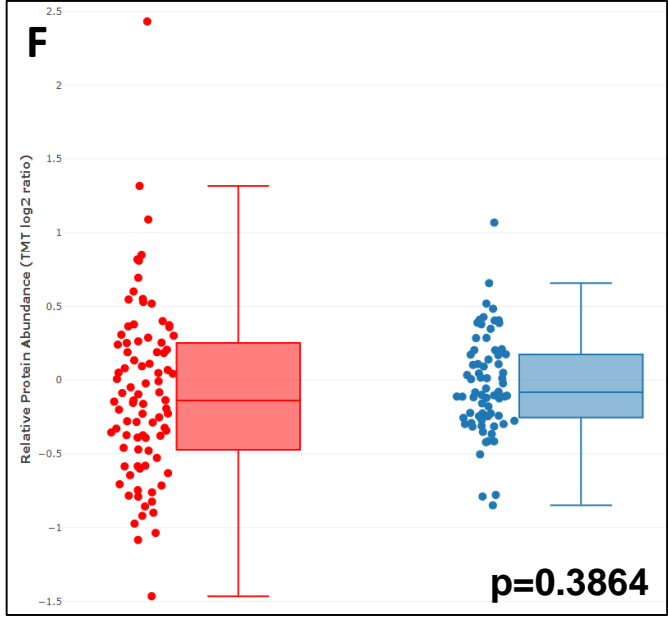

Supplement: Supplementary file 1 [file biomedicines-11-02576-s001.zip › Supplementary Figure S2.pdf]

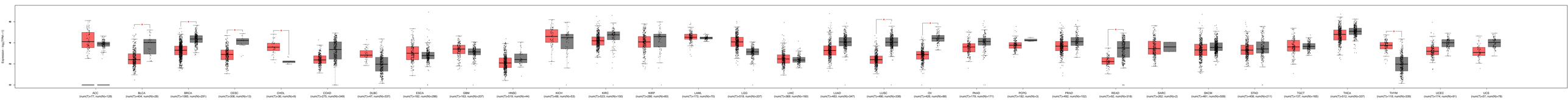

Supplement: Supplementary file 1 [file biomedicines-11-02576-s001.zip › Supplementary Figure S3.pdf]

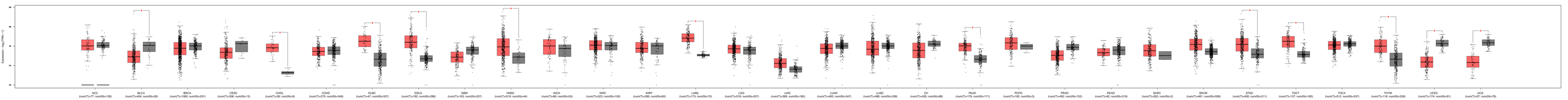

Supplement: Supplementary file 1 [file biomedicines-11-02576-s001.zip › Supplementary Figure S4.pdf]

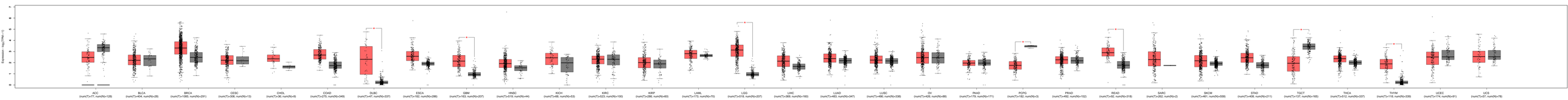

Supplement: Supplementary file 1 [file biomedicines-11-02576-s001.zip › Supplementary Figure S5.pdf]

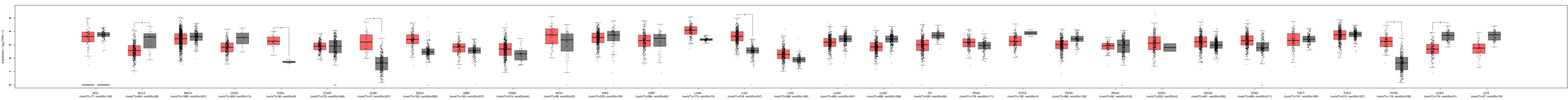

Supplement: Supplementary file 1 [file biomedicines-11-02576-s001.zip › Supplementary Figure S6.pdf]

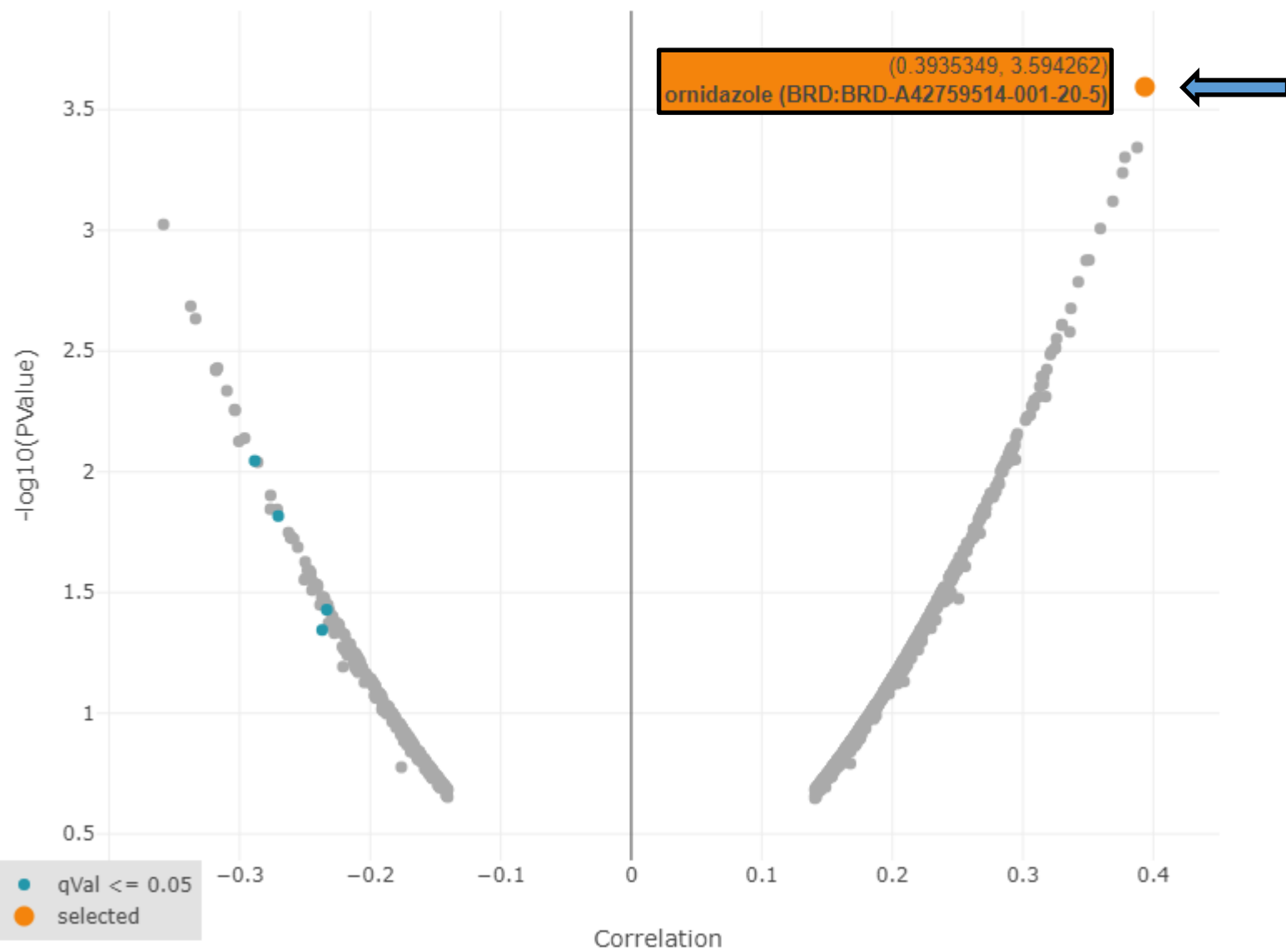

Supplement: Supplementary file 1 [file biomedicines-11-02576-s001.zip › Supplementary Figure S7.pdf]
